# Madras Quarterly Journal of Medical Science

**Published:** 1861-07

**Authors:** 


MADRAS QUARTERLY JOURNAL OF MEDICAL SCIENCE.—We have received the first three numbers of this able periodical, and shall be happy to place it upon our exchange list. Mr. Bohn is the agent for it at London.

